# A Prospective Study to Assess Vancomycin Serum Concentrations inPediatric Patients with Current Dosing Guidelines

**Published:** 2016

**Authors:** Peyman Arfa, Abdollah Karimi, Sedigheh Rafiei Tabatabaei, Alireza Fahimzad, Shahnaz Armin, Mohammad Sistanizad

**Affiliations:** a *Department of Clinical Pharmacy, Faculty of Pharmacy, Shahid Beheshti University of Medical Sciences, Tehran, Iran.*; b *Pediatric Infections Research Center, Mofid Children’s Hospital, Shahid Beheshti University of Medical Sciences, Tehran, Iran*; c *Emam Hossein Medical and Educational Center, Shahid Beheshti University of Medical Sciences, Tehran, Iran.*

**Keywords:** Vancomycin, Pediatrics, Trough Level, Therapeutic Drug Monitoring

## Abstract

Concerns about increasing bacterial resistance to vancomycin, have caused the adult treatment guidelines to recommend higher trough concentrations based on the type and location of infectious disease. Although these recommendations are not specific to children, the values can be extrapolated.

This prospective study was designed to evaluate efficacy of current vancomycin dosing recommendations to achieve therapeutic trough serum concentration in pediatric patients. Laboratory data, vancomycin dosing and subsequent serum concentrations of children in a community teaching pediatrics hospital were collected and analyzed. Trough serum levels were determined at steady state and compared with Infectious Disease Society of America (IDSA)

2011 guidelines for the treatment of Methicillin-Resistant Staphylococcus Aureus (MRSA)

infections.

In a prospective observational, cross-sectional study in a university medical center in Tehran, Iran, 50 patients, who received vancomycin for more than 4 doses, were recruited and their trough vancomycin level was determined. The mean age and creatinine clearance of patients were 5.47 ± 4.24 and 87.5 ± 31.25, respectively. Eleven (22%) patients received vancomycin at 40 mg/kg/day (low dose) and 39 (78%) at 60 mg/kg/day (high dose). Considering trough goals of 10-14 and 15-20 mg/L in low and high dose groups, serum levels in 91% (73% sub- therapeutics) and 85% (69% sub-therapeutics) of patients were not in recommended therapeutic range, respectively.

This study has shown that current recommended vancomycin dosing regimens in pediatric patients (40-60 mg/kg/day), resulted in sub-therapeutic serum concentrations in our study population.

## Introduction

It is becoming increasingly difficult to ignore the vancomycin due to a long history of administration, lower cost and clinical experience, as the gold-standard treatment of infections caused by methicillin-resistant Staphylococcus aureus (MRSA) in pediatric age group and adults both, despite all the limitations,such as poor tissue penetration, adverse effects and relatively slow bacterial killing (1, 2).

During the last decade, utilization of vancomycin has been increased (1). Inappropriate use of vancomycin has not only led to increase in health care costs but also contribute to the emergence of resistant organisms. Recently, Vancomycin-Resistant gram positive organisms is increasingly recognized as a serious, worldwide public health concern. Adjustment and targeting of specific serum concentration of vancomycin above the Minimum Inhibitory Concentration (MIC) for the offended organisms is recommended to avoid bacterial resistance (3).

According to the guideline published by Infectious Disease Society of America (IDSA) in

2011 for assessment of the vancomycin efficacy, the prevention of possible adverse effects such as ototoxicity or nephrotoxicity and also prevention of bacterial resistance, monitoring and adjustment of trough vancomycin concentrations are required in both adult and children. The recommended trough level is 15-

20 mg/L for severe infections such as bacteremia, endocarditis, osteomyelitis, meningitis and hospital-acquired pneumonia caused by MRSA. For other infections the trough level of 10-15 mg/L is advised (2, 4, 5).

Based on current guidelines, in pediatric patients with normal renal function, defined as Glomerular Filtration Rate (GFR) > 60 mL/min, the recommended dose of vancomycin is 40-60 mg/kg/day (5, 6). We undertook this descriptive prospective study to evaluate vancomycin dosing regimen and associated serum concentration in children to determine the ability of vancomycin usual dosing to achieve the recommended therapeutic serum concentrations.

*Study design and setting*

This study was designed as a cross- sectional descriptive, prospective study in a group of pediatric patients receiving vancomycin. The study was carried out between May 2013 and Feb 2014, at Mofid Children Hospital, affiliated to Shahid Beheshti University of Medical Sciences, Tehran, Iran and 50 patients entered to the study. Patients were selected by physician and clinical pharmacist joint decision in regular visits. Ethics approval had been obtained from the ethics committee of Shahid Beheshti University of Medical Sciences.

## Experimental

Patients between one month and 18 years old, who received vancomycin were recruited in the study. The inclusion criterion was receiving at least 3 successive doses of vancomycin (fixed dose and dosing interval of every 6 h). Exclusion criteria were vancomycin dosing with intervals other than the every 6 h, administration of nephrotoxic drugs (including aminoglycosides, loop diuretics, amphotericin B and colistin) concurrently with vancomycin, anuria and dialysis. There were no leaving out criteria for initial analyses based on a disease state or condition. All patients received vancomycin as 1 h infusion to reduce probability of Red Man Syndrome (7, 8).

Informed consent was obtained from the patient’s legally authorized representative before entrance to the study.

*Assessments*

Baseline data recorded were age, sex, height, type of infection and serum creatinine. Also descriptive statistic were used for analyses the information such as vancomycin dosage and administration, duration of infusion, trough serum concentration, time that trough level was sampled and diagnosis. Vancomycin blood level was measured after the drug has reached steady state condition. Administration of at least 3 doses, has been considered as steady state condition (1, 6).Two mL venous blood samples to determine vancomycin trough level were collected from every patient within 15 minutes before administering next dose of vancomycin. The serum was separated by centrifugation (3000 rpm, 10 min), and the isolated serum was immediately frozen at −80 °C.

Serum Creatinine sampling was done at the same day (± day) of vancomycin serum concentration sampling. Schwartz equation was used for estimation of creatinine clearance.(7, 9).

Serum concentrations of vancomycin for vancomycin usage were Central Nervous System (CNS) infections (42%), respiratory infections (20%), skin/soft tissue infections (10%), Fever without localized site (FWLS) (8%), osteomyelitis/septic arthritis (6%) and others (14%).

In order to assess relationship between vancomycin trough concentrations and pediatric references dosing, the patients were divided into 2 groups; low dose and high dose with dosing of 40 mg/kg/day and 60 mg/kg/day, respectively.

Low dose was used for patient with non- severe infections such as skin/soft tissue infection, community-acquired pneumonia and high dose was used in patients with more severe infections such as central nervous system infections, hospital-acquired pneumonia. This division is performed based on current clinical guidelines (4, 5).

The administered dose in low dose group was 37.53 ± 5.71 mg/kg/day (Median: 40) and in high dose group was 59.43 ± 3.05 mg/ kg/day (Median: 60). Eleven and thirty nine patients included in low and high dose groups, were analyzed by Fluorescence Polarization Immunoassay (FPIA) (Cobas Integra 400 system from Roche Diagnostics, Switzerland). The lower detection limit of this assay was 0.74 µg/mL, and the coefficients of variation (CV%) were 3.0% at 8.70 µg/mL, 2.2 at 26.3 µg/mL, and 3.3% at 54.6 µg/mL.

**Table 1:** Patient Demographics and Vancomycin Dosing Characteristics

**Sex n (%)**	
Male	33 (66)
Female	17 (34)
Location n (%)	
Pediatric infectious disease ward	48 (96)
PICU	2 (4)
Age	
Median (range)	5 (4 month-14y)
Mean (SD)	5.47(4.24)
Weight ,kg	
Median (range)	17.5 (3-88)
Mean (SD)	22(17.3)
Serum creatinine, mg/dL	
Mean(SD)	0.65 (0.16)
Creatinine Clearance, mL/min	
Mean (SD)	87.5 (31.25)
Vancomycin dose, mg/kg/day	
Mean (S.D)	53.97 (10.04)
Mode	60
Median (range)	59.34 (23.63-70.58)

*Data analysis*

The Statistical Package for the Social Sciences (SPSS, version 18.0; IBM Company, USA) was used for data analysis. Data was presented as numbers, percentages, mean (SD or 95% confidence interval) unless otherwise stated.

## Results

Fifty four consecutive patients (19 females, 35 males) were enrolled in this study. Four patients were excluded due to discontinuation of vancomycin before fourth dose. The patients’ demographic data and vancomycin dosing data are shown in Table 1.

**Figure 1:**
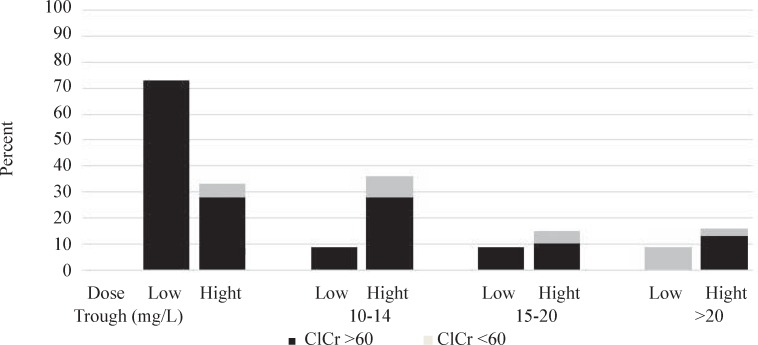
Percent of vancomycin trough levels in two groups (n = 11 in low dose and n = 39 in high dose group) based on Clcr

During our study, the most commonly cited respectively. Additionally, due to impact of renal function on vancomycin clearance and subsequently trough concentration, patients were divided to two groups based on their creatinine clearance; creatinine clearance ≥ 60 mL/min and creatinine clearance ˂ 60 mL/min. Figure 1 shows the vancomycin trough levels in our patients. The trough levels have been divided into four categories: less than 10 mg/L (sub- therapeutic), 10-14 mg/L (therapeutic level for low dose vancomycin), 15-20 mg/L (therapeutic level for high dose vancomycin) and more than 20 mg/L (over-therapeutic level) (1, 4-6).

The results, as shown in Figure 1, considering trough goals of 10-14 and 15-20 mg/L in low and high dose groups, 91% and 85% of patients in low and high dose groups, failed to reach the desired corresponding serum concentration. 73% of patients in low dose group and 69% in high dose group had sub-therapeutic vancomycin drug levels. The percent of patients with vancomycin level of more than 20 mg/L were 9% and 15% in low and high dose groups, respectively. In 73% of patients in low dose group and 33% in high dose group trough levels were below 10 mg/L.

Creatinine clearance in six (15%) patients in high dose group and in one (5%) in low dose group were less than 60 mL/min at entrance to the study. All of these patients received vancomycin every 6 h. Vancomycin blood level just in one of these six patients was more than 20 mg/L.

In a 12 years old obese female patient (weight = 88 kg, Body Mass Index (BMI) = 36 kg/m2) with orbital cellulitis who received vancomycin 880 mg every 6 h, drug-induced nephrotoxicity has occurred. Serum creatinine increased from 0.7 mg/dL to 1.4 mg/dL on the third day of vancomycin treatment. The patient’s serum creatinine normalized 48 after discontinuation of vancomycin. Trough serum concentration of vancomycin was 49 mg/L in this patient. We defined vancomycin-induced nephrotoxicity base on KDIGO practice guideline for acute kidney injury (10).

## Discussion

The present study was designed to determine the ability of pediatric current recommended dosing of vancomycin to achieve therapeutic serum concentration. To our knowledge, our study is one of the few prospective studies in this field. The most interesting finding was that the majority of patients in both low and high dose groups-despite the major difference in the dose of the vancomycin-failed to reach the desired corresponding serum concentration (91% and 85%, respectively). Contrary to expectations, serum vancomycin concentration in the majority of patients who had decreased renal function, were sub therapeutic. This finding was unexpected and emphasizes the importance of therapeutic drug monitoring in drug renal dose adjustment. However, low number of cases with renal dysfunction in our study is major limitation for this conclusion.

Low percent of patients with therapeutic vancomycin level in our study is in agreement with many of other previous studies (1, 6). In a great deal of the previous work in this field, authors analyzed 435 trough serum concentrations in 295 patients. In this study vancomycin dosing was stratified in this manner; less than 40, 40-59, 60-79, and ≥ 80 mg/kg/day. The corresponding trough serum concentration were also divided in to 5 groups; less than 5, 5-9, 10-14, 15-20, and greater than 20 mg/L. Fifty-seven percent of all trough concentration were less than 10 mg/L. Their study showed that recommended vancomycin dosing in children (40-60 mg/kg/day) produced sub therapeutic serum concentration. Authors recommended that vancomycin total daily dose should be increased to 70-85 mg/kg/day (6).

In accordance with the present results, previous pediatric studies have demonstrated that a common vancomycin dosing regimen, 40 mg/kg/day, was not high enough to achieve trough level of over 10 mg/L (1, 3, 6).

In another study, authors evaluated the impact of increasing the recommended vancomycin starting dose from 45 to 60 mg/kg/day on vancomycin trough concentrations in pediatrics (n = 182). They concluded that starting dose of 60 mg/kg/day decreased the likelihood of an initial low vancomycin trough concentration < 5 mg/L (17%) (11), but their study indicated that the 60 mg/kg/day dose did not consistently achieve a vancomycin trough concentration of 15-20 mg/L (11).

Adult’s studies showed that when treating invasive MRSA infection, Area-under-the- concentration-time-curve (AUC) for 24 h divided by the MIC (AUC24/MIC) > 400 for vancomycin is best predictor of treatment outcomes (2, 4, 6, 7, 12). Frymoyer *et al. *using predictor models and a hypothetical population of healthy children evaluated the ability of current vancomycin dosing to achieve this AUC24/MIC ratio (3). This study showed that for MRSA isolates with an MIC 0.5 μg/mL, both doses (40, 60 mg/kg/ day) achieved AUC24/MIC > 400, when MIC was 1.0 μg/mL, the 40 mg/kg/day dose always predicted an AUC24/MIC < 400, For MIC of 2.0 μg/mL, all AUC24/MIC predictions were not above 400 for both doses. Interestingly, when MIC was 1.0 μg/mL in three of four models, the dose 60 mg/kg/day could create enough AUC (3). In our study around 69% of the patients had sub therapeutic vancomycin levels with 60 mg/kg/ day dosing. The correlation between therapeutic levels and AUC/MIC ratio in pediatric, should be reevaluated in clinical field.

Nephrotoxicity is an important concern of vancomycin administration. In a review article, authors evaluated retrospective data from various studies (with total 307 patients). They suggested; Obesity, concomitants nephrotoxins usage, high trough level for long duration and ICU stay (vasopressin use or high APACHEII (Acute Physiology and Chronic Health Evaluation II) score), as risk factors for vancomycin-induced nephrotoxicity (8, 13). In our study, vancomycin- induced nephrotoxicity, is occurred in an obese patient.

Prospective design and similarity with results of other studies are important advantages of our study.

These findings suggest that currently recommended vancomycin dosing in children, should be re-evaluated. Combining these findings with increasing Vancomycin Intermediate Staphylococcus Aureus (VISA) in sub therapeutic vancomycin levels, especially with trough levels below 10 mg/L (4, 6, 14), support the conceptual premise that therapeutic drug monitoring is an important component of treatment in patients receiving vancomycin.

Further research should be done to investigate the other empiric vancomycin dosing regimens concentration.

Re-evaluation of vancomycin dosing regimens in pediatrics and therapeutic drug monitoring with supervision of clinical pharmacy service for rationalizing MRSA pharmacotherapy is recommended.
